# Molecular Characterization of Glucose-6-Phosphate Dehydrogenase: Do Single Nucleotide Polymorphisms Affect Hematological Parameters in HIV-Positive Patients?

**DOI:** 10.1155/2020/5194287

**Published:** 2020-08-01

**Authors:** Kwabena Owusu Danquah, Kofi Mensah, Charles Nkansah, Samuel Kwasi Appiah, Mark Noagbe, Yasmine Hardy, David O. Ntiamoah, Lillian Antwi Boateng, Max Efui Annani-Akollor, Eddie-Williams Owiredu, Alexander Yaw Debrah, Otchere Addai-Mensah

**Affiliations:** ^1^Department of Medical Diagnostics, Faculty of Allied Health Sciences, Kwame Nkrumah University of Science and Technology, Kumasi, Ghana; ^2^Department of Haematology, Komfo Anokye Teaching Hospital, Kumasi, Ghana; ^3^HIV Clinic, Komfo Anokye Teaching Hospital, Kumasi, Ghana; ^4^Department of Basic and Applied Biology, University of Energy and Natural Resource, Sunyani, Ghana; ^5^Department of Molecular Medicine, School of Medicine and Dentistry, Kwame Nkrumah University of Science and Technology, Kumasi, Ghana

## Abstract

This descriptive, cross-sectional study aimed at evaluating the prevalence of G6PD deficiency and the 376A ⟶ G, 202G ⟶ A single nucleotide polymorphisms (SNPs) among HIV patients attending care at a teaching hospital in Ghana and determine how the SNPs affect haematological profile in HIV. A total of 200 HIV-positive Ghanaians were recruited. Venous blood samples were obtained and complete blood count, and G6PD screening and genotyping for the 376A ⟶ G, 202G ⟶ A SNPs were performed. Out of the 200 participants, 13.0% (26/200) were G6PD-deficient based on the methemoglobin reductase technique, with 1.5% (3/200) and 11.5% (23/200) presenting with partial and full enzyme defect, respectively. Among the 13.0% participants with G6PD deficiency, 19.2% (5/26), 30.8% (8/26), and 19.2% (5/26) presented with 376A ⟶ G only (enzyme activity (EA): 1.19 U/g Hb), 202G ⟶A only (EA: 1.41 U/g Hb), and G202/A376 SNPs (EA: 1.14 U/g Hb), respectively. Having the 376A ⟶ G mutation was associated not only with lower red blood cell (RBC) count (3.38 × 10^6^/*µ*L (3.16–3.46) vs 3.95 × 10^6^/*µ*L (3.53–4.41), *p* = 0.010) but also with higher mean cell volume (MCV) (102.90 (99.40–113.0) vs 91.10 fL (84.65–98.98), *p* = 0.041) and mean cell haemoglobin (MCH) (33.70 pg (32.70–38.50) vs 30.75 pg (28.50–33.35), *p* = 0.038), whereas possessing the 202G ⟶ A mutation was associated with higher MCV only (98.90 fL (90.95–102.35) vs 91.10 fL (84.65–98.98), *p* = 0.041) compared to G6PD nondeficient participants. The prevalence of G6PD deficiency among HIV patients in Kumasi, Ghana, is 13.0% prevalence, comprising 1.5% and 11.5% partial and full enzyme defect, respectively, based on the methemoglobin reductase technique among HIV patients in Ghana. Among G6PD-deficient HIV patients, the prevalence of G202/A376 SNPs is 19.2%. The 376A ⟶ G mutation is associated not only with lower RBC count but also with higher MCV and MCH, whereas the 202G ⟶ A mutation is associated with higher MCV compared to the normal G6PD population.

## 1. Introduction

Human immunodeficiency virus (HIV) is a chronic viral infection and a serious public health concern. Currently, approximately 37.9 million people are living with HIV worldwide [1]. In Ghana, 330,000 people are living with HIV [2].

HIV infection is associated with persistent inflammation and immune activation leading to production of reactive oxygen molecules and oxidative stress [3, 4]. Additionally, HIV-positive individuals are predisposed to a plethora of other infections, which may result in oxidative stress. The sequelae of these oxidative stresses are particularly alarming and life-threatening in people comorbid with glucose-6-phosphate dehydrogenase (G6PD) deficiency. These complications may include acute hemolytic anemia, which can result in cardiovascular, renal, liver, and other organ system complications [5]. Furthermore, red blood cell hemolysis in G6PD deficiency can lead to methemoglobinemia. The prevalence rate of G6PD deficiency is 5–25% in tropical Africa and Asia [5–7]. In Ghana, the prevalence of G6PD deficiency is 15–26% [8, 9].

Over 400 G6PD variants have been identified [10], and the polymorphisms are predominantly defined to specific geographic locations [11]. About 186 of these variants are associated with G6PD deficiency due to the decreasing enzyme activity or stability [5, 12, 13]. In sub-Saharan Africa, the predominant G6PD variants are *B*, *A*, and *A-*, with frequencies greater than 1% [14]. The G6PD *B* variant possesses the 376A cDNA sequence and has been shown to have a normal enzyme activity. Likewise, the G6PD *A* variant, which carries a cDNA mutation A376G, has about 85% of the normal enzyme activity. On the contrary, the G6PD *A-* variants carry the G6PD *A* backbone with an added single nucleotide mutation. The most common G6PD *A-*variant possesses the A376G/G202A mutation and has been reported to have 10% of the normal enzyme activity in their red blood cells (RBC), although their white blood cells (WBC) maintain 100% of the normal enzyme activity [15]. Other *A-* variants peculiar to sub-Saharan Africa are A376G/T968C, A376G/G680T, and A376G/A543T [16].

In some conditions such as malaria, before primaquine administration, G6PD deficiency is screened. However, the advantage of screening HIV-positive patients for G6PD deficiency is often overlooked despite reports indicating worse clinical outcomes in people comorbid with HIV and G6PD deficiency [17–19]. Importantly, HIV and G6PD deficiency have individually being linked with deranged hematological profile. HIV affects all hematological cell lines, as evidenced by anemia, neutropaenia, lymphopaenia, and thrombocytopaenia [20–23], whereas G6PD deficiency is associated with attenuated levels of haemoglobin (Hb), haematocrit (HCT), mean cell volume (MCV), and mean cell haemoglobin (MCH) [24]. Notwithstanding, studies on G6PD deficiency in HIV patients is limited in Africa, where both conditions are prevalent, and none has been conducted in Ghana.

This study, thus, aimed at evaluating the prevalence of G6PD deficiency and the 376A ⟶ G and 202G ⟶ A single nucleotide polymorphisms (SNPs) among HIV patients attending care at a teaching hospital in Ghana and determine if the SNPs are associated with deranged hematological profile.

## 2. Materials and Methods

### 2.1. Study Design/Area

This descriptive, cross-sectional study was carried out between June 2018 and May 2019 at the HIV clinic of Komfo Anokye Teaching Hospital (KATH) in Kumasi.

### 2.2. Study Population

The sample size for the study was calculated using Fischer's sampling formula (*N* = *Z*^2^*PQ*/*d*^2^), where *Z* is the critical value of the normal distribution (1.96 at 95% CI); *P* is the estimated prevalence of G6PD deficiency in Ghana (15%) [8]; *d* is the absolute precision; or sampling error tolerated = 5%. From the above equation, a total of 250 consecutive consenting HIV-positive Ghanaians, aged 15 years and above, were invited to partake in the study during their routine clinic visit days. All participants were on ART. Fifty (50) participants were either on sulfate and copper containing medications, were very ill or pregnant, and were exempted from the study. A total of 200 HIV-positive patients were thus included in the analysis.

### 2.3. Sample Collection and Assay

Six milliliters (6 ml) of venous blood were obtained from each participant under aseptic conditions for laboratory assessments. Complete blood count was evaluated using an XN 2000 fully automated Sysmex haematology analyzer (Sysmex Corporation, Kobe, Japan). G6PD screening was performed with the methemoglobin reductase technique as described by Brewer et al. [25], and patients were grouped into “normal”, “partial defect,” and “full defect” based on the color of the test solution as described by Antwi-Baffour et al. [26] (Details in Table S1). The G6PD enzyme activity assay was performed for samples that were G6PD-deficient (both “full” and “partial defect”) during screening by the methemoglobin reductase technique using the Pointe Scientific G6PD kinetic kit according to manufacturer's instructions (standardized with an intra-assay % CVs of 2.5%–9.2% and interassay %CVs of 2.1%–11.4%) (Pointe Scientific Limited, UK). In preparation for G6PD genotyping, DNA was extracted from the blood samples that were G6PD-deficient during screening. Extraction was based on the double salt precipitation method as previously described [27]. A large number of single nucleotide polymorphisms (SNPs) have been identified to be associated with G6PD deficiency in Africa [10, 11, 16]. However, 376A ⟶ G and 202G ⟶ A SNPs are the most commonly reported in Ghana [14, 28] and were thus selected for this study. For the 376A ⟶ G mutation, the forward and reverse primer sequences used were 5′-CCCAGGCCACCCCAGAGGAGA-3′ and 5′-CGGCCCCGGACACGCTCATAG-3′, respectively, whereas those for the 202G ⟶ A mutation were 5′-CACCACTGCCCCTGTGACCT-3′ and 5′-GGCCCTGACACCACCCACCTT-3′, respectively (Inqaba Biotech Ltd, South Africa). The PCR cycling conditions were as follows: one cycle of initial denaturation at 94°C for 5 minutes, denaturation at 94°C for 45 seconds, annealing at 56°c for 30 seconds, and extensions at 74°C for 45 seconds followed by five cycles of final extension at 74°c for 5 minutes (for 35 cycles). The amplified products were separated by electrophoresis on 1% agarose gels stained with ethidium bromide and visualized under UV light for the presence of bands indicative of 376A ⟶ G and 202G ⟶ A mutations (Figure S1).

### 2.4. Ethics Approval and Consent to Participate

This study was approved by the Committee on Human Research Publication and Ethics (CHRPE) of the School of Medical Sciences and Kwame Nkrumah University of Science and Technology. Written informed consent was obtained from all participants who opted to participate after the aims and objectives of the study were explained to them.

### 2.5. Statistical Analysis

Statistical analysis and graphical presentation were performed using the R Language for Statistical Computing version 3.5.2 (R Core Team, Vienna, Austria) [29]. Categorical data were presented as frequencies (percentages). Normality of continuous data was evaluated using Shapiro–Wilk's test. All continuous data were nonparametric and were presented as medians (interquartile ranges). Significance of differences of hematological parameters between various variants of G6PD were tested with the Kruskal–Wallis tests, followed by Dunn's post hoc multiple comparison tests. All statistical tests were two-sided, and a *p* value < 0.05 was considered statistically significant.

## 3. Results

A total of 200 participants with an average age of 42.0 (35.0–50.0) years were included in this study. A higher proportion was females (84.0%) and had basic education (65.5%). The average RBC count, Hb, MCV, MCH, platelet (PLT) count, and WBC count were 3.88 × 10^6^/*µ*L, 12.0 g/dL, 91.75 fL, 31.25 pg, 222.0 × 10^3^/*µ*L, and 5.13 × 10^3^/*µ*L, respectively (Table 1).

**Figure 1:**
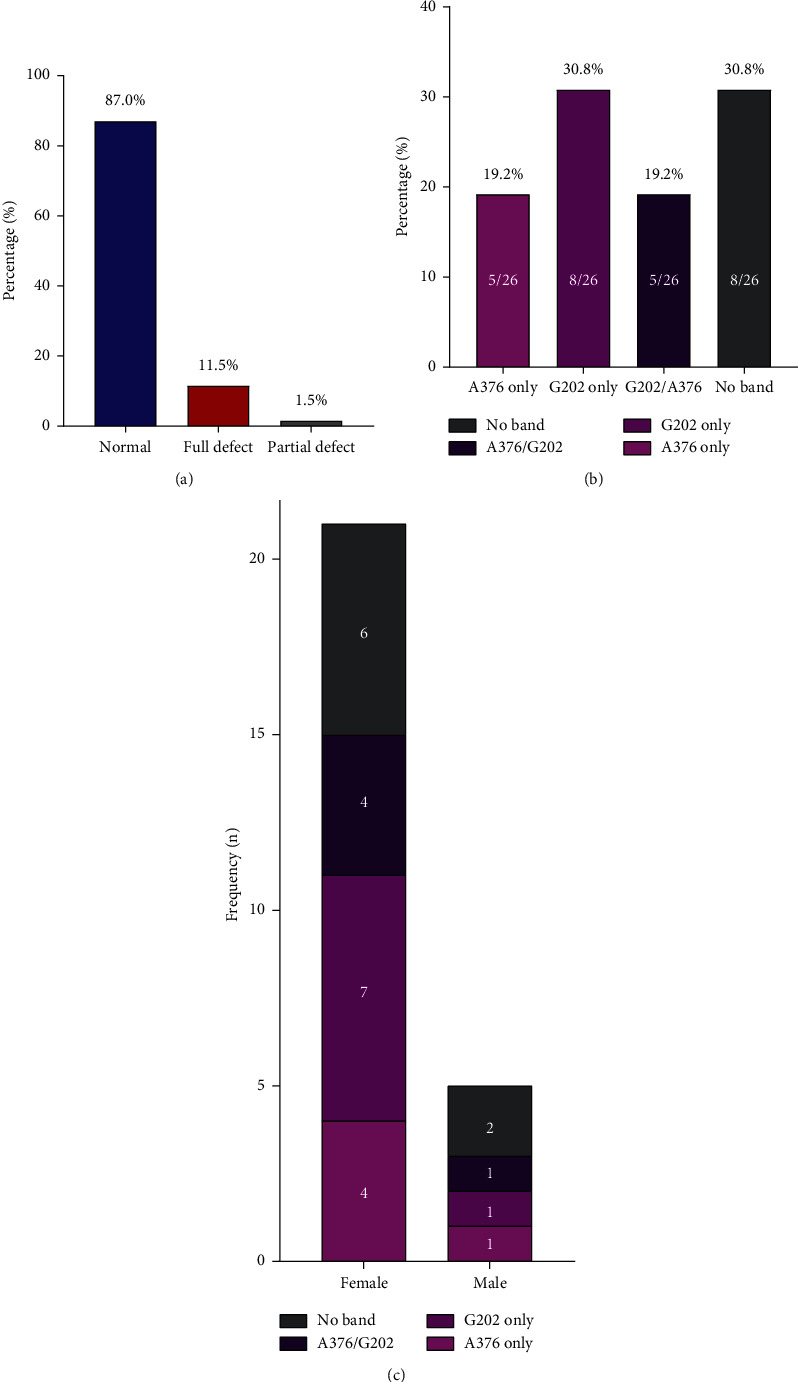
Prevalence of G6PD deficiency. (a) Phenotypic prevalence of G6PD deficiency based on the methemoglobin reductase technique. Percentages were calculated over the total population (*n* = 200). (b) Genotypic prevalence of 376A ⟶ G, 202G ⟶ A, and G202/A376 G6PD variants. Percentages were calculated over the number of G6PD-deficient participant (*n* = 26). (c) Genotypic prevalence of 376A ⟶ G, 202G ⟶ A, and G202/A376 G6PD variants by sex.

Out of the 200 participants screened using the methemoglobin reductase technique, 13.0% (26/200) were G6PD-deficient, with 1.5% (3/200) and 11.5% presenting with partial and full enzyme defects, respectively (Figure 1(a)). Among the 13.0% (26/200) with G6PD deficiency, 19.2% (5/26), 30.8% (8/26), and 19.2% (5/26) presented with 376A ⟶ G only, 202G ⟶ A only, and G202/A376 SNPs, respectively (Figure 1(b)). Upon stratification by sex, 4, 7, 4, and 5 of the females and 1, 1, 1, and 2 of the males had the 376A ⟶ G only and 202G ⟶ A only, G202/A376 SNPs, and no band, respectively (Figure 1(c)). The likelihood of being phenotypically classified as G6PD full defect was higher among participants having the G202/A376 SNPs compared to the 202G ⟶ A SNP (Table S2). Furthermore, participants with the 376A ⟶ G had a relatively lower G6PD enzyme activity compared to those with the 202G ⟶ A SNP, although not statistically significant (Figure S2).

**Figure 2:**
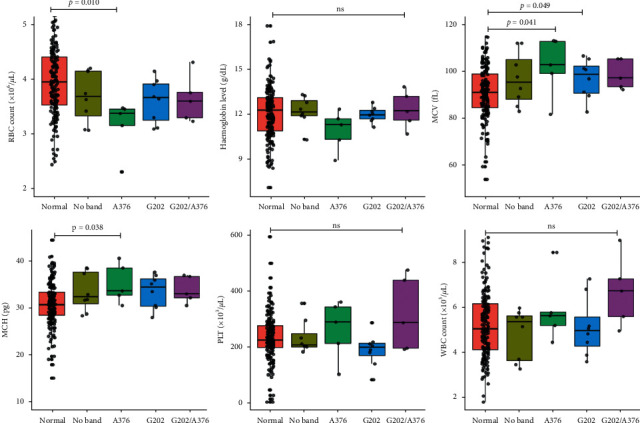
Comparison of hematological parameters by G6PD genotype. RBC: red blood cell, MCV: mean cell volume, MCH: mean cell haemoglobin, PLT: platelet, WBC: white blood cell, and ns: not significant. Data presented as medians (interquartile ranges). Significance of differences of hematological parameters between variants of G6PD were tested with the Kruskal–Wallis tests and Dunn's post hoc multiple comparison tests.

Participants with only the 376A ⟶ G mutation presented not only with significantly lower RBC count (3.38 × 10^6^/*µ*L (3.16–3.46) vs 3.95 × 10^6^/*µ*L (3.53–4.41), *p* = 0.010) but also with higher MCV (102.90 (99.40–113.0) vs 91.10 fL (84.65–98.98), *p* = 0.041) and MCH (33.70 pg (32.70–38.50) vs 30.75 pg (28.50–33.35), *p* = 0.038) compared to G6PD-nondeficient participants. On the other hand, participants with only the 202G ⟶ A mutation had significantly higher MCV (98.90 fL (90.95–102.35) vs 91.10 fL (84.65–98.98), *p* = 0.041). No statistically significant association was found between haemoglobin level, PLT, and WBC counts and G6PD variants (Figure 2).

## 4. Discussion

This study reports a 13.0% prevalence of G6PD deficiency, comprising 5% and 11.5% partial and full enzyme defect, respectively, based on the methemoglobin reductase technique. As expected, the prevalence of G6PD deficiency in this study was lower than a report by Adu et al. [8] and Owusu et al. [30] who indicated an overall prevalence of 19.5% and 19.3%, respectively, among prospective blood donors and pregnant women in Ghana. Correspondingly, in another study among the general population in Nigeria, Okafor et al. reported a prevalence of 17.78% [31]. Contrarily, a study in Uganda by Roh et al. [32] found a lower prevalence of G6PD deficiency (8.6%) among the general population. Apart from disparities in study populations (HIV vs general population), differences in the test methods could account for the variations in prevalence rates. Our finding is, however, consistent with a retrospective study by Tungsiripat et al. among HIV patients in the United States. After screening 212 Blacks infected with HIV, they found 28 (13.2%) to be G6PD-deficient [33]. In another study by Serpa et al. [34] in the United States, 6.8% of all HIV-infected adults had G6PD deficiency, which is lower compared to this present study. The disparity in the prevalence rates could be linked to the fact that Serpa et al. included participants of diverse race (African Americans, Hispanics, Whites, and Asian-Pacific). Evidence suggests that the prevalence of G6PD deficiency is very low among Whites compared to Blacks [33, 35]. Thus, the inclusion of Caucasians may have attenuated the prevalence rate found in their study. Our finding also falls within the prevalence range of 5–25% found in tropical Africa, the Middle East, tropical and subtropical Asia, some parts of the Mediterranean, and in Papua New Guinea [5–7].

In this study, among the participants with G6PD deficiency, we found 19.2% to harbor the G6PD *A*- allele (G202/A376), which is associated with the reduced enzyme activity [15]. A study by Xu et al. in the Dominican Republic also reported a similarly high prevalence of the G6PD *A*- variant among HIV-infected patients [36]. Of note, we also observed that, among those with G6PD deficiency based on the methemoglobin reductase technique, 30.8% presented with no band on electrophoresis. It is possible that these patients harbored other G6PD variants such as the A376G/T968C, A376G/G680T, and A376G/A543T, which are also peculiar to sub-Saharan Africa [16]. Importantly, seven and four of the females versus one and two of the males had the G6PD *A* and G6PD *A*- allele, respectively. Congruently, more females than males with the G6PD *A*- variants have been reported in previous studies in Ghana [14, 28]. An explanation could be the higher number of females in this study compared to males. The consistently higher number of HIV-positive females compared to males in Kumasi justifies the gender disparity [37, 38].

Another finding of this study is that the presence of only the 376A ⟶ G mutation was associated not only with lower RBC count but also with higher MCV and MCH, whereas possessing only the 202G ⟶ A mutation was only associated with significantly higher MCV. The relatively greater deranged haematological profile in the participants with the 376A ⟶ G compared to the 202G ⟶ A could be attributed to the comparatively lower G6PD enzyme activity among participants with the 376A ⟶ G mutation compared to the 202G ⟶ A mutation, although not statistically significant. In a study to find the association between G6PD deficiency and hematological parameters in children from Botswana, Motshoge et al. made similar observations [39]. The increased MCV due to the 202G ⟶ A mutation is also in harmony with a GWAS study by Ding et al. [40]. Other reports such as those by Ajlaan [41] and Domingos et al. [42] are in line with our study findings.

Clinically, HIV infection is linked with chronic inflammation, which is associated with increased oxidative stress [3, 4]. Furthermore, HIV predisposes the infected person to other infections such as malaria, which may induce oxidative stress [43, 44], and administration of primaquine as treatment could aggravate the already existing oxidative stress. Moreover, trimethoprim-sulfamethoxazole, a commonly used medication in HIV can precipitate hemolysis. These suggest that HIV patients who are G6PD-deficient are at higher risk of life-threatening oxidative stress-induced complications if they are not identified. It is thus important for HIV patients to be screened for G6PD deficiency. Indeed, guidelines for G6PD screening have been shown to prevent the omission or oversight for later testing when oxidant drugs are administered on an urgent or emergent basis and have been linked with reduced risk of complications associated with G6PD deficiency in HIV patients [33].

### 4.1. Limitations

Unavailability of data on important modifying genotypes such as HbS; alpha-thalassemia 3.7 deletion, as well as data on ART; its adherence; and CD4 count is a limitation of this study. This study is also limited by the relatively small sample size and the cross-sectional design used, which precluded comparison with the non-HIV group. Larger sample sizes in future studies will be ideal.

## 5. Conclusion

This study reports a 13.0% prevalence of G6PD deficiency, comprising 1.5% and 11.5% partial and full enzyme defect, respectively, based on the methemoglobin reductase technique among HIV patients in Ghana. Among G6PD-deficient HIV patients, the prevalence of G202/A376 SNPs is 19.2%. The 376A ⟶ G mutation is associated not only with lower RBC count but also with higher MCV and MCH, whereas the 202G ⟶ A mutation is associated with higher MCV compared to the normal G6PD population.

## Figures and Tables

**Table 1:** Baseline characteristics of the study population.

Variables	Frequency (*n* = 200)	Percentage (%)
Demographic		
Sex		
Female	168	84.0
Male	32	16.0
Age (years)^∗^	42.0	35.0–50.0
Educational level		
Illiterate	44	22
Basic	131	65.5
Secondary	19	9.5
Tertiary	6	3.0
Hematological^∗^		
RBC count (×10^6^/*µ*L)	3.88	3.47–4.34
Hb (g/dL)	12.00	10.97–13.10
MCV (fL)	91.75	85.05–100.0
MCH (pg)	31.25	28.80–34.33
PLT count (×10^3^/*µ*L)	222.0	196.80–591.00
WBC count (×10^3^/*µ*L)	5.13	4.83–6.17

^∗^Data are presented as median and interquartile ranges.

## Data Availability

The data used to support this study are included within the article (and its supplementary information files).

## Supplementary Materials

Supplementary Materials Table S1: procedure for G6PD screening by the methemoglobin reductase technique. Table S2: comparison between genotypic and phenotypic G6PD deficiency prevalence. Figure S1: gel image depicting a SNPs. Figure S2: comparison of the G6PD enzyme activity by the presence of 376A ⟶ G only, 202G ⟶ A only, and G202/A376 SNPs.
