# Supplementary material for: Molecular Characterization of Glucose-6-Phosphate Dehydrogenase: Do Single Nucleotide Polymorphisms Affect Hematological Parameters in HIV-Positive Patients?
Source: J Trop Med. 2020 Aug 1;2020:5194287. doi: 10.1155/2020/5194287 (PMC7416277; doi:10.1155/2020/5194287)
Supplement: Supplementary Materials — Table S1: procedure for G6PD screening by the methemoglobin reductase technique. Table S2: comparison between genotypic and phenotypic G6PD deficiency prevalence. Figure S1: gel image depicting a SNPs. Figure S2: comparison of the G6PD enzyme activity by the presence of 376A ⟶ G only, 202G ⟶ A only, and G202/A376 SNPs. [file 5194287.f1.docx]

# **Molecular characterization of Glucose-6-Phosphate Dehydrogenase: do single nucleotide polymorphisms affect hematological parameters in HIV positive patients?**

**Table S1. Procedure for G6PD screening by the methemoglobin reductase technique**

| 1. Three test tubes labeled (T) for test, (P) for positive control and (N) for negative control were used for each individual test. 2. 1ml of blood was dispensed into tube (T) containing 50µl of sodium nitrite and 50µl of methylene blue; 1ml of blood was dispensed into tube (P) containing 50µl of sodium nitrite and 1ml of blood was dispensed into tube (N) containing 50µl of methylene blue. 3. The test tubes were incubated at 37°C for 3 hours with hourly mixing interval. 4. After the incubation period, three test tubes were arranged and labelled with their corresponding labels as in the initial tubes and 0.1ml of physiological saline was dispensed into each tube 5. 0.1ml of the respective incubated samples was transferred into the tubes and color of each mixture was compared. 6. The test results were recorded as G6PD normal when the color of the test solution was similar to the red color of the negative control. G6PD full defect was recorded when the color of the test solution was similar to the brown color of the positive control and as partial G6PD defect when the color was midway between normal G6PD activity and G6PD full defect. |
| --- |


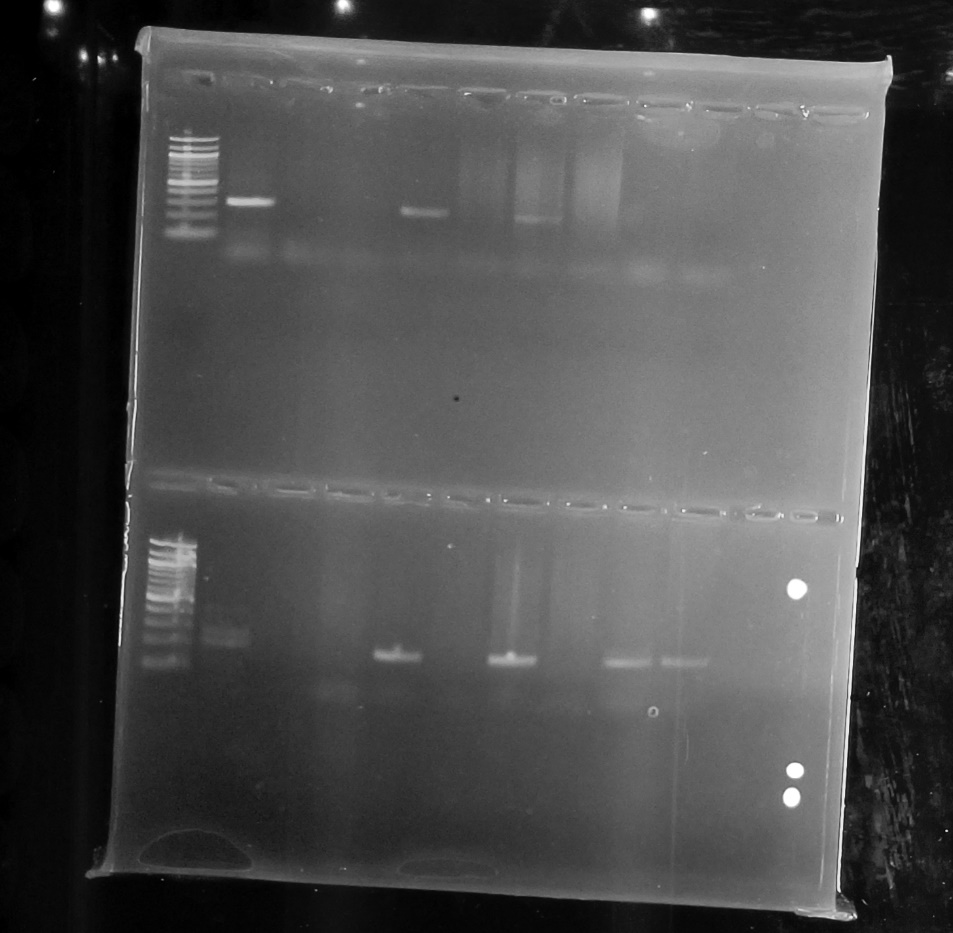


**311bp**

**L 9 8 7 6 5 4 3 2 1 C**

**L 9a 8a 7a 6a 5a 4a 3a 2a 1a C**

**100bpppp**

**208bp**

**Figure S1: Gel image depicting a SNPs**: L=ladder; C=Negative controls; lane1-9=G202A samples; lane1a-9a =A376G samples; lane 1,2,4,6,8,9=positive bands G202A;4a,6a,9a=positive bands A376G

**Table S2. Comparison between genotypic and phenotypic G6PD deficiency prevalence**

| Genotype | Phenotype | | OR (95% CI) | p-value |
| --- | --- | --- | --- | --- |
|  | Partial defect | Full defect |  |  |
| G202 | 1 | 7 |  |  |
| A376 | 2 | 3 | 0.21 (0.01-3.37) | 0.273 |
| G202/A373 | 0 | 5 | 2.20 (0.07-64.91) | 0.648 |
| No band | 0 | 8 | 3.40 (0.12-96.71) | 0.716 |


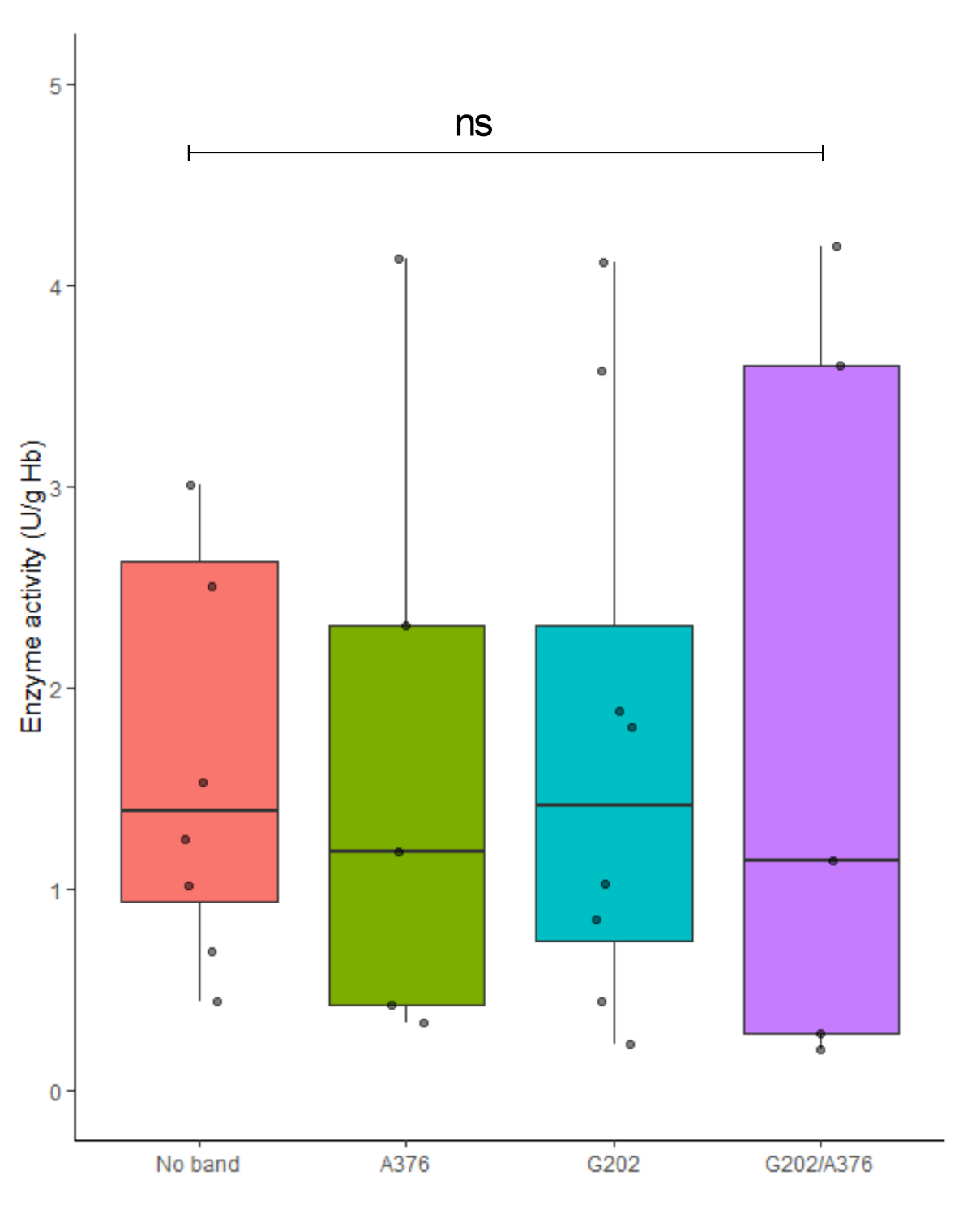


**Figure S2. Comparison of G6PD enzyme activity by the presence of 376A → G only and 202G → A only, and G202/A376 SNPs.**

The average G6PD enzyme activity for participants with 376A → G only, 202G → A only, and G202/A376 SNPs were 1.19 U/g Hb (0.43-2.31), 1.41 U/g Hb (0.74-2.31), and 1.14 U/g Hb (0.28-3.60), respectively. No significant differences were observed between G6PD enzyme activity with respect to G6PD variants (**Fig. S2**).
